# Pattern of distribution and etiologies of Midline diastema among Kurdistan-region Population

**DOI:** 10.4317/jced.57122

**Published:** 2020-10-01

**Authors:** Hasan-Sabah Hasan, Arkan-Muslim Al Azzawi, Ayshan Kolemen

**Affiliations:** 1 Specialist orthodontist at orthodontic department of khanzad polyclinic teaching center / General directorate of hawler / ministry of health/ Kurdistan region- Iraq; 2 Specialist orthodontist at orthodontic department/ Babylon university/ college of dentistry

## Abstract

**Background:**

Aim of study was to evaluate the prevalence and etiological factors that contribute in midline diastema in Kurdistan region-Iraq population among different age groups and genders.

**Material and Methods:**

Orthodontic patients sample of (EX: 1021orthodontic patients (537 males and 484 females) were randomly selected from Kurdistan-region population, attending to orthodontic department of khanzad polyclinic teaching center (General directorate of hawler / Ministry of health/ Kurdistan region- Iraq) during 2018-2019 period. Aged (13-35 years) with mean age ± SD was 19.6 ± 4.8 years, with a median of 19 years. The examination included patient history taking, intraoral examination, photograph, intraoral periapical radiography of incisors area and panoramic radiographic. Diastema consider positive when the space between central incisors is 0.5mm and more, width was measured clinically used digital Vernier calipers at one millimeter above the incisors edge.

**Results:**

The prevalence of midline diastema was 23.2%. located in the maxilla (97%), in mandible (1.3%) and in both was (1.7%). The prevalence of midline diastema differs significantly between the age groups (*p*< 0.001). The highest prevalence (55.8%) was among patients aged ≥ 30 years, and it was also high (37.7%) among those aged < 15 years. The prevalence among females (26.4%) was significantly higher than the prevalence (20.3%) among males (*P*= 0.020). The main causes of midline diastema in females was thumb sucking and missing lateral incisors (14.1% and 12.5% respectively) and in males was high labial frenum and super numerally teeth (39.4% and 30.3% respectively).

**Conclusions:**

Prevalence of diastema in Kurdistan regional- Iraq area was (23.2%), the location mostly in maxilla (97%). The prevalence of diastema in females more than males. The main causes of diastema in females was thumb sucking and missing lateral incisors while in the males was high labial frenum and super numerally teeth.

** Key words:**Prevalence, midline diastema, high labial frenum, thumb sucking.

## Introduction

Diastema in Greek means interval, gap or space between two or more adjacent teeth. Spacing of upper or lower central incisors is commonly known as midline diastema. It has been defined as a natural spacing between the central incisors occurring more frequently on the upper teeth (1). Improve facial aesthetics is one of the main reasons why patients are addressing the orthodontist, facial symmetry having a determining importance in facial aesthetics. Face symmetry and midline coordination are essential criteria for achieving harmony and facial balance.

According to Proffit, (2009) midline diastema was (6.0%) in adolescents and adults. Keene, (1963) was found the prevalence of midline diastema in the full permanent dentition amounting to (14.8%) in maxilla, and (1.6%) in case of mandible diastema. But according to Lavelle, (1970) maxillary midline diastema incidence in adult’s amounts from (1.6%) to (25.0%). Median diastema founded more frequently in the maxillary than mandibular arch (5), and it could be accompanied by general diastemata (4). Epidemiological studies show that the highest percentage is observed in Africans race in comparison to Caucasians race (3,6).

Regarding the etiological factors of midline diastema, most of clinical researchers believe that not only one cause but multiple factors may contribute to a midline space, allot of etiological factors could be contributed to midline diastema (high frenum attachment, parafunctional habits, supernumerlly teeth (mostly Mesiodens), teeth-jaw discrepancy, missing lateral incisors, disrupted eruption of canine and hereditary family history) (7-13).

Proper treatment of midline diastema need a very well diagnosis of the etiology of midline diastema and a relevant to the specific etiological factor is necessary. Timing of the treatment is important to achieve satisfactory results with long sTable (14-16).

Now a day, we have a lack of information in the literature on the prevalence and etiology of midline diastema in Kurdistan Region-Iraq. The main purpose of this research was to evaluation and investigation the prevalence and etiological factors of midline diastema in Kurdistan Region-Iraq population among different age groups and genders.

## Material and Methods

A sample of (EX: 1021 orthodontic patients (537 males and 484 females) were randomly selected, attending to orthodontic department of khanzad polyclinic teaching center in erbil (General directorate of hawler / Ministry of health/ Kurdistan region- Iraq) during the period extended from 2018-2019 year. Orthodontic patient age was (13-35 years) with mean age ± SD was 19.6 ± 4.8 years, with a median of 19 years, this is the preferred age range for orthodontic treatment.

The examination was include taking patient’s history (including family, trauma, medical and dental history), intraoral examination using direct vision or using dental mirror in some cases under dental chair light source, intraoral periapical radiography of incisors area, panoramic radiography (especially with that related to ectopic eruption of canine or missing lateral incisors), photography in cases related to identification of midline pathology. The examination all was done by same authors. Patients with visible space between maxillary or mandibular central incisors were clinically examined by measuring the width with digital Vernier calipers at 1 mm above the incisors edge. Present of a 0.5mm or more space between the maxillary or mandibular central incisors was considered as positive diastema patient.

The causative factors { Supernumerary teeth (Mesiodens), Missing lateral incisors, High labial frenum, Peg shaped of laterals, Parafunctional habits (Mouth breathing; Tongue thrusting; Lip biting; Thumb sucking,), Macroglossia, Microdontia, Familial characteristic, Dento-alveolar disproportion, Ectopic maxillary canine, midline pathology and Ankylosed central incisors} were identified via intraoral examination including patients / family history, periapical and/ or panoramic radiographs and photography were taken to correlate the clinical findings, Findings of clinical examination of the patients that fulfilled the study criteria were recorded in a specially designed preformat. Positive diastema patients were asked about the presence of diastema in their family members, were they concerned about its presence and will they be opting to treat it in future. The following included criteria was selected in this study:

1. Patient should be at Permanent dentition stage.

2. Both permanent maxillary and mandibular central incisors should be present.

And the most criteria that had excluded was:

1. Extraction teeth.

2. Any periodontal diseases.

3. Any Dento-facial deformity.

4. Previous orthodontic treatment.

5. Ages under 13years and above 35 years.

The statistical Data were analyzed using the Statistical Package for Social Sciences (SPSS, version 22), chi square test of association was used to compare proportions.

Fisher’s exact test was used when the expected count of more than 20% of the cells of the Table was less than 5. A *p* value of ≤ 0.05 was considered statistically significant.

## Results

The study involved (EX: 1021 orthodontic patients (537 males and 484 females). Their mean age ± SD was 19.6 ± 4.8 years, with a median of 19 years. The age range was 13 to 35 years old.

**Figure 1:**
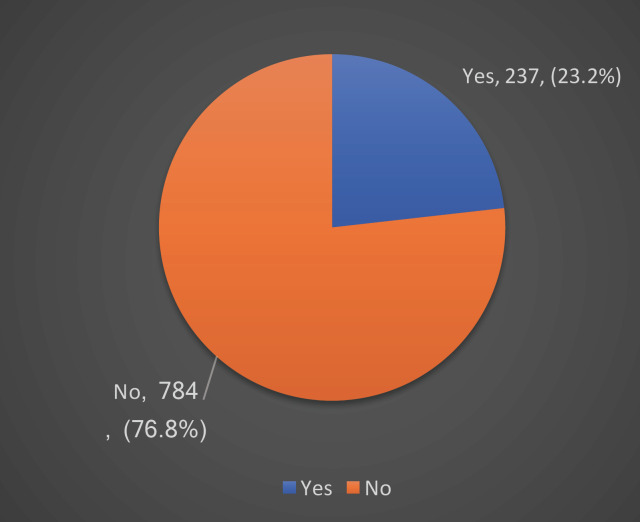
Pie chart showing Prevalence of diastema among Erbil population.

Table 1, shows that the highest proportion (39.7%) of the sample aged 15-19 years, and 33.5% aged 20-24 years. It also shows that 52.6% of the sample were males. The prevalence of diastema was 23.2% as presented in Figure 1.

**Table 1:**
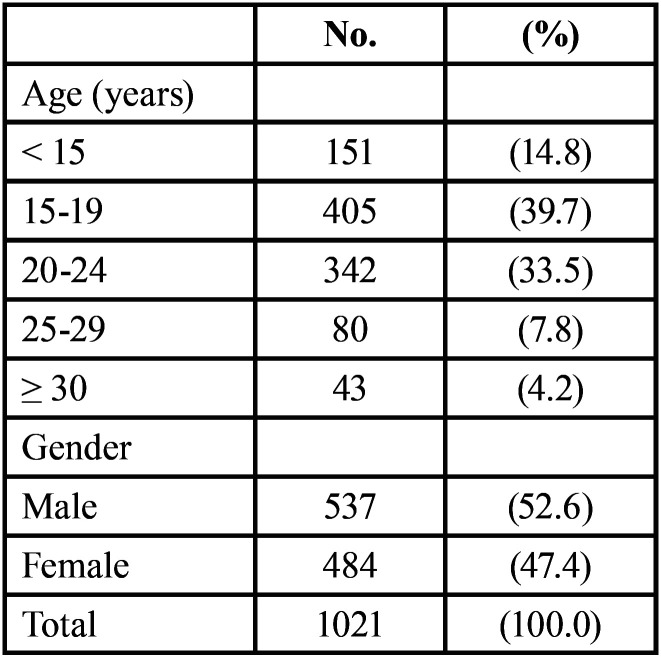
Distribution of sample by age and gender.

It is evident in Table 2, that in almost all the patients the location of the diastema was in the maxilla, either alone (97%) or in both the maxilla and mandible (1.7%).

**Table 2:**
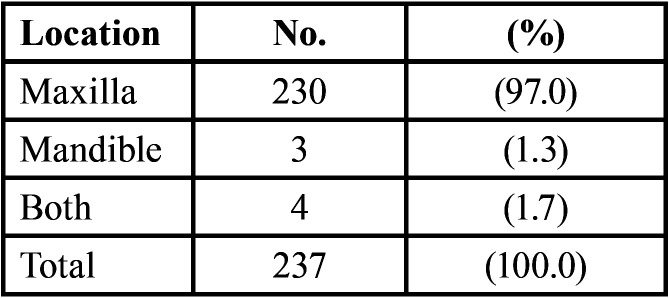
Location of the midline diastema.

Table 3 show that the prevalence of diastema differs significantly between the age groups (*p* < 0.001). The highest prevalence in this study (55.8%) was among patients aged ≥ 30 years, and it was also high (37.7%) among those aged < 15 years, while relatively low rates (< 20%) were observed among patients aged 15-29 years. So there is no consistent pattern. The prevalence of midline diastema in this study among females (26.4%) was significantly higher than the prevalence (20.3%) among males (*p* = 0.020).

**Table 3:**
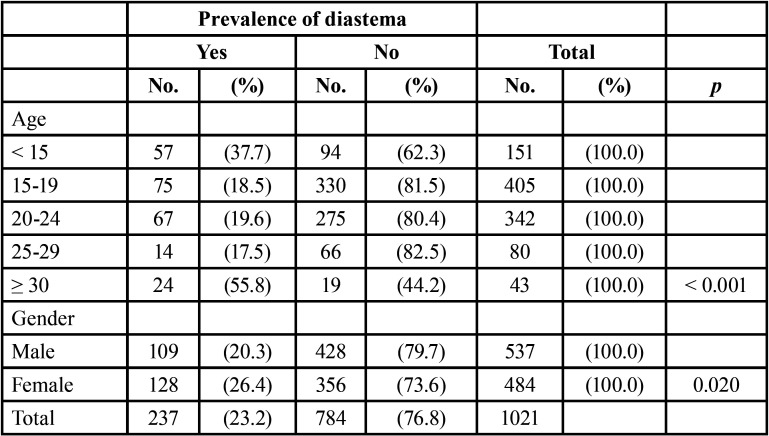
Prevalence of diastema by age and gender.

Table 4 show the main causes of midline diastema as follows: high labial frenum (34.2%), super numerally teeth (23.6%), thumb sucking (10.5%), missing lateral incisors (10.1%), peg shape lateral incisors (6.3%) and Dento-alveolar disproportion (4.6%). The other rare causes are presented in Table 4.

**Table 4:**
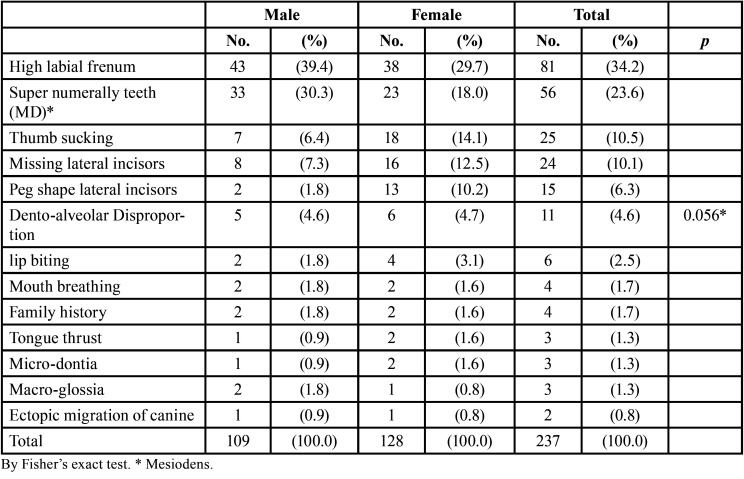
Etiological factors of diastema among the gender.

Nearly significant association was detected between the causes of diastema and gender (*P*= 0.056). It is worth to mention that the proportions of males with high labial frenum (39.4%) and super numerally teeth (30.3%) were higher than those of females (29.7% and 18.0% respectively), while the proportions of thumb sucking and missing lateral incisors among females (14.1% and 12.5% respectively) were higher than those among males (6.4% and 7.3% respectively).

## Discussion

Aim of this study was to determine the prevalence and etiological factors contributed to midline diastema { Supernumerary teeth (Mesiodens), Missing lateral incisors, High labial frenum, Peg shaped of laterals, Parafunctional habits (Mouth breathing; Tongue thrusting; Lip biting; Thumb sucking), Macroglossia, Microdontia, Familial characteristic, Dento-alveolar disproportion, Ectopic maxillary canine, midline pathology and Ankylosed central incisors}, in different age groups and genders among Kurdistan region-Iraq. Ages ranged from (13 - 35 years) is the preferred age range for orthodontic treatment of midline diastema, patients under 13 years were excluded to avoid cases of midline diastema due to the normal stages of development (Ugly daggle stage), patients above 35 years were excluded to avoid cases of midline diastema due to migration of teeth and progressive periodontal diseases (17).

The prevalence of diastema in this study was 23.2%. The prevalence among females (26.4%) was significantly higher than that among to male (20.3%) (*P*= 0.020), this prevalence was considerably same as found in Saudi nationalities (23%) (18), and less than found in Kuwait (26.8%) (19), Baghdad City (28%) (20), adolescent Nigerian (37%) (21), but more than founded in Turkish population (4.5%) (22), United Kingdom of Caucasian (3.4%) (3), Pakistan (12.59%) (23), and south India (1.6%) (24), this differences in findings could be attributed to the increased number of factors contributing to midline diastema or to genders, race differences and hereditary factors.

Location of midline diastema was founded respectively (maxillary 97%, mandibular 1.3%, and in both the maxilla and mandible 1.7%). This could be comparable with that result found in Baghdad City (maxillary 22.5%, mandibular 2.3% and both arches 3.2%) (20), in Tanzanians population, they found the incidence to be (26%, 11% and 8% for maxillary, mandibular, and both respectively) (25), this difference in the midline diastema location could be related to the difference in inclusion criteria, population races, sampling technique or genetic predisposition factors.

In this study, prevalence of diastema was differing significantly between the age groups (*P* < 0.001). The highest prevalence was (55.8%) among patients aged ≥ 30 years, and it was also high (37.7%) among those aged < 15 years. Comparable with that found in Saudi Arabia (20-25 years) (18) and in Jammu papulation (20-24years) (27). This results could be attributed to that; the midline diastema spacing could be reduced through the mesial drifting of permanent teeth following the eruption of third molars. This could explain the reduction in the frequency of midline diastema in ages (25 year) (18).

The prevalence among female’s patients (26.4%) was significantly higher than the male patients (20.3%) (*P*= 0.020) regarding this study, similar to that found in Pakistan population (23) and to that in Baghdad City (26). But differ than found in Saudi Arabia (18), Jammu population (27) (EX: the incidence of male was more than female), this finding may be attributed to the high level of cosmetic concern in that female’s gender predisposing them to visit the orthodontist more frequently or could be attributed to genetic, family factors or hereditary (23,26).

Regarding etiological contributing factors responsible in the development of midline diastema that have been widely reported and discussed in the literature, but till now There is no agreement that single factors could be the precise etiological factor (16).

Regarding this study, the most common etiology effecting (39.4%) of subjects was high labial frenum attachment, this finding came similar to that found by Gupta *et al.*, (27) and Ferguson *et al.*, (31) and Ross *et al.*, (32). The worth mansion frequency of high frenal attachment earlier theory that it might be the cause of diastema but now a day this fact is not free of controversies that high frenum isn’t the main cause but could be contributed to maxillary midline diastema (28,29), next causative factor came (30.3%) was Mesiodens supernumerlly tooth among males, thumb sucking and missing lateral incisors among females (14.1% and 12.5% respectively), this might comparable with that found by Luqman *et al.*, (18), AL-Zahrani, (32) and HamedullahJan *et al.*, (23).

As previously mentioned, there is many factors that contributed in midline diastema such as hypo-dontia, macro-glossia, anchylose central incisors, dentoalveolar discrepancy and ectopic canine eruption. Midline alveolar bone clefts also could be consider as contributing factors of midline diastema (34,35). The difficulties for orthodontist is whether trying to close, open or redistribute the space. Closing space by orthodontist may eliminates the need for prosthetic rehabilitation but may there is an aesthetics or/and function problems especially in case of missing lateral incisors. This was depending on many factors such as amount of over-jet, lip support status, crown color, shape and root position. If all these factors are unfavorable, so opening space and prosthetic replacement will be the best choice (33).

Most important, that the exacted etiological factor of midline diastema was difficulty to detected either in this study or other. Future studies need to detect the exacting single etiological factors correlation of midline diastema with a more purified sampling technique (Individual frequency of some observed etiological factor could be the reasons for this. Furthermore, Bolton’s discrepancy, extractions, variable size of pre-maxilla and periodontal problems must be taken into consideration (23).

## Conclusions

- In this study the prevalence of diastema in Kurdistan Region-Iraq area was (23.2%).

- The location of the diastema was in the maxilla (97%), in mandible (1.3%) and in both was (1.7%).

- The highest prevalence of diastema (55.8%) was among patients aged ≥ 30 years, and it was also high (37.7%) among those aged < 15 years.

- The prevalence among females was (26.4%) significantly higher than that in males (20.3%).

- Main causes of diastema in females was thumb sucking and missing lateral incisors

- The main causes of diastema in males was high labial frenum and super numerally teeth.
